# Effect of unfolded protein response on the immune infiltration and prognosis of transitional cell bladder cancer

**DOI:** 10.1080/07853890.2021.1918346

**Published:** 2021-06-30

**Authors:** Xiaokai Yan, Min Chen, Chiying Xiao, Jiandong Fu, Xia Sun, Zuohuai Hu, Hang Zhou

**Affiliations:** Department of Oncology, the Second Affiliated Hospital of Zunyi Medical University, Zunyi, China

**Keywords:** Transitional cell bladder cancer, unfolded protein response, tumour immune infiltration, tumour progression, prognostic model

## Abstract

**Background:** Bladder cancer (BC) is one of the most common human malignancies worldwide. Previous researches have shown that the unfolded protein response (UPR) pathway could contribute to the tumorigenesis of BC. However, the role of UPR in the immune infiltration, progression, and prognosis of BC is unclear.

**Methods:** The GSVA and ssGSEA methods were used for assessing the UPR score and immune cells infiltration score in three BC public datasets, respectively. The relationship between the UPR pathway and clinicopathological characteristics was analyzed by the Kruskal-Wallis, Wilcox test, and log-rank test. The association of the UPR pathway with various tumor-infiltrating immune cells was evaluated with the correlation analysis. Univariate Cox regression analysis was performed to identify risk factors significantly associated with prognosis. The predictive models were built based on risk factors and visualized with nomograms. The performance of our models was evaluated with the calibration curve, Harrell's concordance index (c-index), and receiver operating characteristic (ROC) analysis.

**Results:** We found that the UPR pathway and many UPR-related genes were significantly associated with the pathologic grade, tumor type, and invasive progression of transitional cell bladder cancer (TCBC), and a high UPR score predicted a poor prognosis in patients. The UPR score was positively correlated with the infiltration abundance of many tumor immune cells in TCBC. Besides, we constructed predictive models based on the UPR score, and good performance was observed, with c-indexes ranging from 0.74 to 0.87.

**Conclusions:** Our study proved that the UPR pathway may have an important impact on the progression, prognosis, and tumor immune infiltration in TCBC, and the models we built may provide effective and reliable guides for prognosis assessment and treatment decision-making for TCBC patients.

## 1. Introduction

Bladder cancer (BC) is the top 10 most common and prevalent human malignancies worldwide, with a yearly estimated 540,000 new cases and 200,000 deaths, and a 5-year age-standardized relative survival rate ranging from 60 to 80% between individual countries, seriously threatening people’s lives and health [1–3]. Transitional cell bladder cancer (TCBC) is the most common subtype of BC. The mainstream view is that the main risk factors affecting the BC patients’ prognosis include pathologic grade, stage, histologic type, lymphovascular invasion, lymph node metastasis and treatment decisions [4]. With the explosion of high-throughput technologies, such as microarray and next-generation sequencing, tens of thousands of genes can be tested simultaneously, greatly prompting unprecedented progress in the research of cancer pathogenesis and identification of prognostic markers within the past 10 years. At present, many previous studies have reported a large number of gene markers in BC, and constructed different prognostic prediction models based on one or several biomarkers [5,6]. However, the actual development of the tumour is an extremely complicated multi-step process, putting aside factors such as the social economy and medical intervention, the progression and outcome of cancer should be attributed to massive genetic abnormality rather than to just one or several markers. Under these conditions, an increasing number of studies are devoted to identifying gene sets (or pathways) according to the biological characteristics of various genes to understand the molecular mechanisms of carcinogenesis, which is becoming an important part of tumour research [7].

The Molecular Signatures Database (MSigDB) is one of the most widely used and comprehensive databases of gene sets for performing gene set enrichment analysis [8]. As a part of MsigDB, hallmark gene sets were developed to convey specific biological states or processes [9]. Previous studies have shown that many hallmark gene sets demonstrated excellent predictive values in the prognosis, response to chemotherapy and progression of some cancers [10–13]. The unfolded protein response (UPR) is a highly conserved signal transduction pathway, mainly including three arms: IRE1, PERK and ATF6, which can drive the expression of genes needed for vigorous protein production and participate in the process of tumour progression [14,15]. Under endoplasmic reticulum (ER) stress conditions, the UPR signalling can lead to cell death if cells cannot overcome the stress conditions, while some cancer cells use the UPR signalling as a selective adaptive advantage for proliferation, survival, and avoiding apoptosis [16,17]. In breast cancer, the UPR signalling was considered to promote a malignant phenotype and resistance to various therapies [18]. In Qi et al.’s [19] research, the UPR signalling was identified as a novel prognostic signature in cutaneous melanoma. Schardt et al. [20] found that activation of the UPR signalling in acute myeloid leukaemia was associated with particular clinical characteristics and a more favourable course of the disease. Besides, the UPR pathway was closely related to the immune response, and showed crucial functions in the development, differentiation, function and survival of various immune cells [21–24]. Owing to the important role of the immune response in tumorigenesis and tumour progression [25–28], it is necessary to explore the impact of the UPR pathway in tumour immune infiltration.

Currently, Chia et al. [29] have demonstrated that the UPR signalling could contribute to the tumorigenesis of BC, but the role of UPR in the immune infiltration, progression, and prognosis of BC is unclear. This study attempts to clarify the impact of UPR on the immune response and outcome of BC using bioinformatics methods and clinical analysis, which may be helpful to further reveal the underlying pathological mechanism of BC, discover new prognostic markers and guide clinical treatment decisions.

## 2. Materials and methods

### 2.1. Data acquisition and processing

Three BC gene expression datasets (GSE13507, GSE5287 and GSE1827) were downloaded from the Gene Expression Omnibus (GEO) (https://www.ncbi.nlm.nih.gov/geo). The data processing methods were the same as our previous research [30]. The GSE13507 dataset contains nine normal bladder mucosae, 58 bladder mucosae surrounding cancer, 165 TCBC and 23 recurrent tumour samples. The clinicopathological characteristics of the 165 TCBC samples can be acquired according to the prompt of Kim et al. [31]. The prognostic information of GSE5287 and GSE1827 was gained from PRECOG (https://precog.stanford.edu), and the other clinicopathological characteristics can be roughly understood in Als et al. [32] and Blaveri et al.’s [33] researches. The GSE5287 includes 30 TCBC samples, and the GSE1827 contains 74 TCBC and 6 squamous cancer samples. For the inability to get detailed pathological information of GSE1827, the squamous cancer samples cannot be removed.

### 2.2. Hallmark gene sets, immune cells infiltration, stromal and tumour purity score

The hallmark gene sets were scored with the Gene Set Variation Analysis (GSVA) method [34,35]. The immune cells infiltration score was assessed *via* the single sample gene set enrichment analysis (ssGSEA) method [36–39]. The stromal and tumour purity score were evaluated through the ESTIMATE algorithm [40–43].

### 2.3. Identification of key gene set

The Kruskal–Wallis or Wilcox test was performed for differential detection of 50 hallmark gene sets in different tissues (Normal bladder mucosae, bladder mucosae surrounding cancer, TCBC and recurrent tumour samples), tumour types (NMIBC, non-muscle invasive bladder cancer; MIBC, muscle invasive bladder cancer), tumour progression (with or without non-muscle/muscle invasive progression) and tumour grades in GSE13507. A *p*-value < .01 or *p*-value < .05 (two-sided) was considered to indicate statistical significance. The UPR score was significantly associated with the tissue type, tumour type, tumour progression and tumour grade, so we selected it for further analysis. Besides, we further analysed the genes in the UPR gene set to identify the master regulators associated with BC.

### 2.4. The effect of UPR on the prognosis

165 TCBC patients were divided into two groups by the median UPR score (0.0304612), and the relationship between the UPR score and patient prognosis (OS, overall survival; CSS, cancer specific survival; PFS, progression-free survival) was analysed by log-rank test. The prognostic impact of the UPR score was validated in the GSE5287 and GSE1827 datasets, and X-tile (version 3.6.1, Yale University School of Medicine) was used to select the best cut-off value of UPR score for grouping [44]. A *p*-value <0.05 (two-sided) was considered to show statistical significance.

### 2.5. The effect of UPR on immune infiltration

The correlation analysis was performed to evaluate the association of UPR score with various tumour infiltrating immune cells. GSE5287 and GSE1827 datasets were used as external cohorts to further validate the effect of UPR on immune infiltration. A *p*-value < .05 (two-sided) was considered to show statistical significance.

### 2.6. Construction and validation of the prognostic models based on UPR

Univariate Cox regression analysis was performed to identify risk factors significantly associated with OS, CSS and PFS. A *p* < .05 (two-sided) was considered statistically significant. The prognostic models were built based on risk factors and visualised with nomograms. Calibration abilities of the prognostic models were tested with calibration plots using 1000 bootstrap resamples. Meanwhile, Harrell’s concordance index (c-index) and receiver operating characteristic (ROC) analysis were performed to validate the predictive ability of the prognostic models.

### 2.7. Statistical analysis

All data processing and statistical analyses were done with R (https://www.r-project.org/, v 3.6.0). The heat-maps were plotted with the R package “pheatmap”. The box plots were made with the R package “ggplot2”. The survival curves and Cox regression analysis were carried out on the R packages “survival” and “survminer”. The correlation heat-map was drawn with the “corrplot” package. The calibration plots, c-index and nomograms were performed with the R package “rms”. The ROC curves were plotted using the R package “qROC”.

## 3. Results

### 3.1. Differential detection of the hallmark gene sets

**Figure 1.**
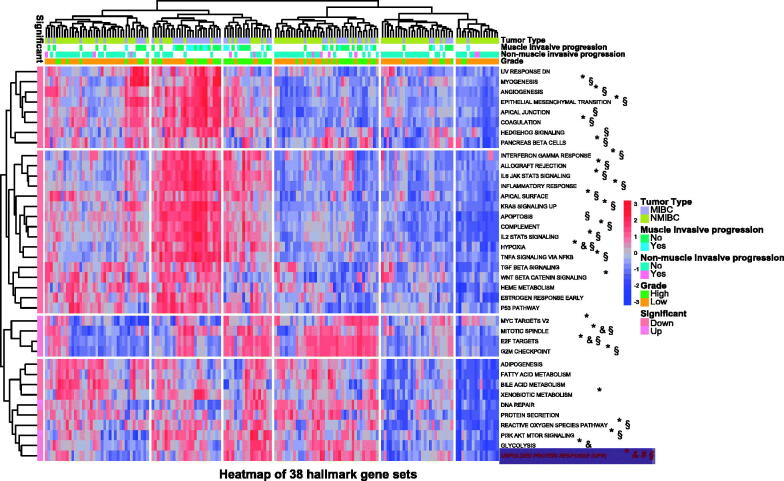
Differential detection of 38 hallmark gene sets in different tumour types, muscle invasive progression, non-muscle invasive progression and tumour grades. NMIBC: non-muscle invasive bladder cancer; MIBC: muscle invasive bladder cancer; TCBC: transitional cell bladder cancer; down: low score in TCBC and recurrent tumour samples; up: high score in TCBC and recurrent tumour samples; **^§^***p* < .05 in NMIBC and MIBC; **^#^***p* < .05 in patients with and without muscle invasive progression; ^&^*p* < .05 in patients with and without non-muscle invasive progression; *p < .05 in low and high tumour grades.

**Figure 2.**
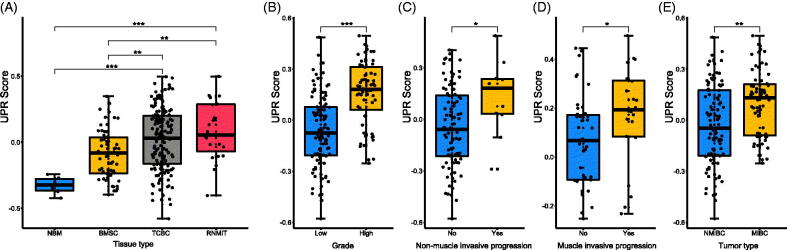
Relationship between unfolded protein response (UPR) and clinic-pathological information. (A) The UPR score in different tissue types; (B) The UPR score in low and high tumour grades; (C) The UPR score in patients with and without non-muscle invasive progression; (D) The UPR score in patients with and without muscle invasive progression; (E) The UPR score in different tumour types. BMSC: bladder mucosae surrounding cancer; MIBC: muscle invasive bladder cancer; NBM: normal bladder mucosae; NMIBC: non-muscle invasive bladder cancer; RNMIT: recurrent non-muscle invasive tumour; TCBC: transitional cell bladder cancer. *p < .05; **p < .01; ***p < .001.

Thirty-eight hallmark gene sets showed the difference in the normal bladder mucosae, bladder mucosae surrounding cancer, TCBC and recurrent tumour samples, including the UPR pathway, E2F targets, G2M checkpoint, P53 pathway, Wnt/beta-catenin signalling etc (Supplementary Figure S1). The UPR score was significantly associated with the tumour type, muscle invasive progression, mon-muscle invasive progression and tumour grade of TCBC (Figure 1). Previous studies have demonstrated that the UPR signalling has crucial functions in the development, progression and survival of many cancers, but the role of it in the immune infiltration, progression and prognosis of BC is unclear. Therefore, we selected it for further analysis. According to the analysis results, the UPR score was higher in the tumour tissues (TCBC and recurrent tumour samples) (Figure 2(A)), samples with high grade (Figure 2(B)), samples with non-muscle/muscle invasive progression (Figure 2(C,D)) and MIBC samples (Figure 2(E)) (*p* < .05). Besides, we analyzed all the 113 UPR-related genes to identify the master regulators. About 53 (46.9%) genes were differentially expressed in the different tissue types, and 40 (35.4%) genes were associated with the tumour type, invasive progression or tumour grade (Supplementary Figure S2). For example, the expression of EIF4EBP1 was higher in the tumour tissues (Supplementary Figure S3A), MIBC (Supplementary Figure S3B) and high-grade samples (Supplementary Figure S3C).

**Figure 3.**
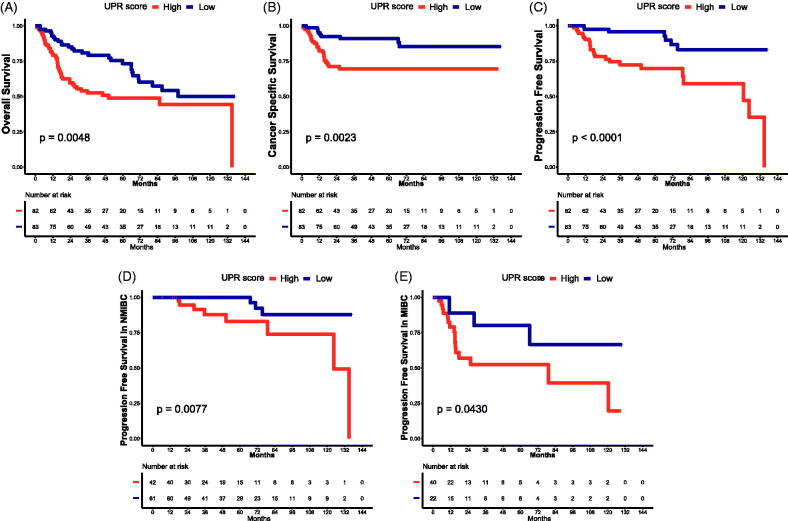
Survival analysis of UPR. Survival curves of UPR score for OS (A), CSS (B), PFS (C), PFS in NMIBC (D) and PFS in MIBC (E). CSS: cancer-specific survival; OS: overall survival; PFS: progression-free survival; MIBC: muscle invasive bladder cancer; NMIBC: non-muscle invasive bladder cancer.

### 3.2. The effect of UPR on the prognosis of TCBC

To assess the impact of UPR on the prognosis, we divided 165 TCBC patients into low and high UPR score groups. According to the results of survival analysis, the high UPR score was significantly associated with poor OS (Figure 3(A)), CSS (Figure 3(B)) and PFS (Figure 3(C)) (*p* < .05). Besides, when we examined the progression in NMIBC and MIBC, respectively, the time to progression was shorter in patients with a high UPR score than a low UPR score (Figure 3(D,E)) (*p* < .05).

### 3.3. The relationship of UPR and the TCBC immune infiltration

**Figure 4.**
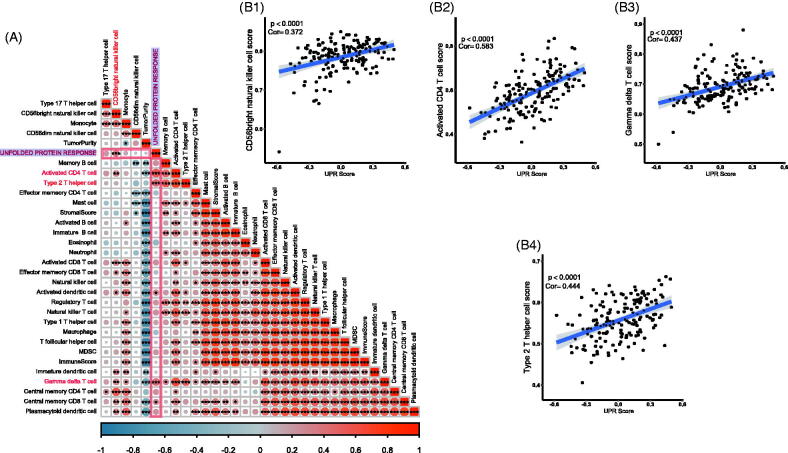
Relationship of UPR and TCBC immune infiltration. (A) Correlation heat map of the UPR score, 28 tumour infiltrating immune cells, stromal score and tumour purity; (B1) Correlation of UPR and CD56bright natural killer cell; (B2) Correlation of UPR and activated CD4 T cell; (B3) Correlation of UPR and gamma delta T cell; (B4) Correlation of UPR and type 2 T helper cell. *p < .001; **p < .0001; ***p < .00001; Cor: Pearson's correlation coefficient.

Because the immune infiltration is deeply involved in cancer progression and prognosis, we tested the correlation among UPR score, 28 tumour infiltrating immune cells, stromal score and tumour purity. The results showed that the UPR pathway may be associated with the infiltration of activated CD4 T cell, type 2 T helper cell, gamma delta T cell, CD56bright natural killer cell, activated dendritic cell and central memory CD8 T cell in tumour tissues (Figure 4(A)) (*p* < .001). However, no clear statistical correlation between the UPR pathway and stromal score/tumour purity was observed (*p* > .05).

### 3.4. External validation of the role of UPR in TCBC

**Figure 5.**
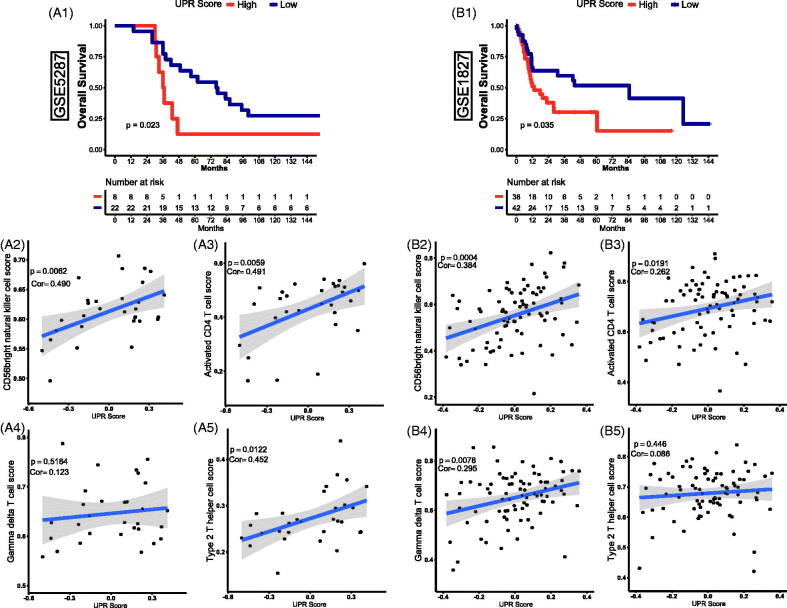
External validation of the effect of UPR on prognosis and immune infiltration in TCBC. (A1, B1) Survival curves of UPR score for OS in GSE5287 and GSE1827; (A2, B2) Correlation of UPR and CD56bright natural killer cell in GSE5287 and GSE1827; (A3, B3) Correlation of UPR and activated CD4 T cell in GSE5287 and GSE1827; (A4, B4) Correlation of UPR and gamma delta T cell in GSE5287 and GSE1827; (A5, B5) Correlation of UPR and type 2 T helper cell in GSE5287 and GSE1827; Cor: Pearson's correlation coefficient.

In accordance with the analysis above in the GSE13507 dataset, the UPR pathway was significantly related to the progression, prognosis, and immune infiltration of TCBC. To further clarify the role of the UPR pathway, the GSE5287 and GSE1827 datasets were used as externally validated cohorts. Through survival analysis, we found that a high UPR score was also significantly associated with a poor OS (Figure 5(A1,B1)) (*p* < .05). Besides, the activated CD4 T cell, type 2 T helper cell, gamma delta T cell and CD56 bright natural killer cell was significantly correlated with the UPR score (Figure 4(B1–B4)) (*p* < .0001), so we selected them for further validation in the external cohorts. Through correlation analysis, the CD56b right natural killer cell and activated CD4 T cell were also showed a positive correlation with the UPR score (Figure 5(A2,A3,B2,B3)) (*p* < .05). Similar relevance of type 2 T helper cell and gamma delta T cell was also observed in GSE5287 and GSE1827, respectively (Figure 5(A5,B4)) (*p* < .05). However, although the correlation trend of gamma delta T cell in GSE5287 (Figure 5(A4)) (Pearson's correlation coefficient, Cor = 0.123) and type 2 T helper cell in GSE1827 (Figure 5(B5)) (Cor = 0.086) was similar to the results in GSE13507, the statistically significant p values were not observed (*p* > .05).

### 3.5. Construction and validation of the prognostic models based on UPR

**Figure 6.**
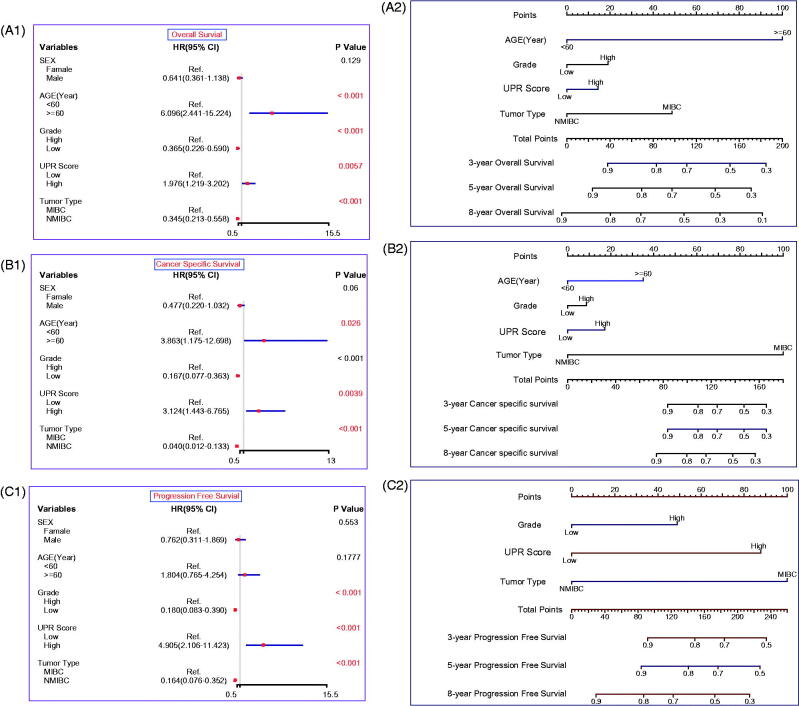
Construction of the prognostic models. Univariate Cox regression analysis for OS (A1), CSS (B1) and PFS (C1). Nomograms for OS (A2), CSS (B2) and PFS (C2). Nomogram total points (OS) = Age (<60 years, score 0; ≧60 years, score 1) × 100 + Grade (Low grade, score 0; High grade, score 1)×19 + UPR score (Low UPR score, score 0; High UPR score, score 1) × 14 + Tumour type (NMIBC, score 0; MIBC, score 1) × 49; Nomogram total points (CSS) = Age × 35 + Grade × 9 + UPR score × 17 + Tumour type × 100; Nomogram total points (PFS) = Grade × 49 + UPR score × 88 + Tumour type × 100.

**Figure 7.**
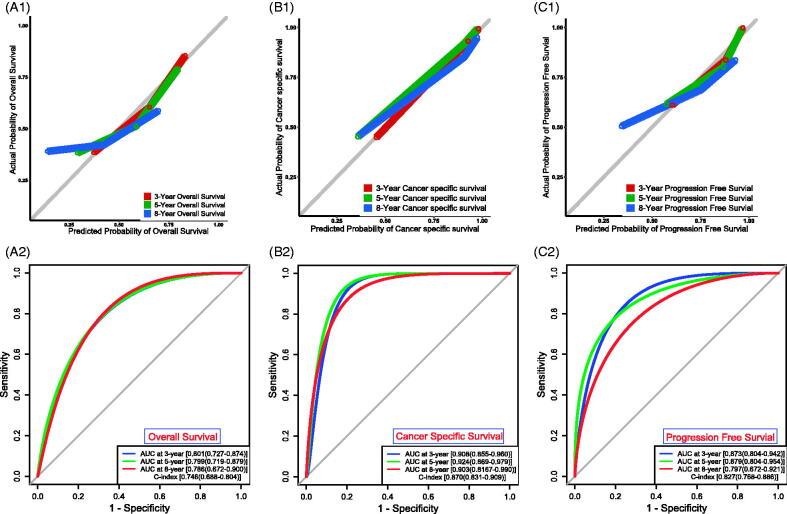
Assessment of the prognostic models. Calibration plots for OS (A1), CSS (B1) and PFS (C1). ROC curves and C-indexes for OS (A2), CSS (B2) and PFS (C2).

To build applicable and individualised prognostic nomograms, we performed the univariate Cox analysis to identify risk factors for OS, CSS and PFS. According to the results, we chose the UPR Score, age, grade and tumour type to construct nomograms for OS (Figure 6(A1,A2)) and CSS (Figure 6(B1,B2)), and the UPR Score, grade and tumour type were selected for PFS (Figure 6(C1,C2)). Then the calibration plots for OS (Figure 7(A1)), CSS (Figure 7(B1)) and PFS (Figure 7(C1)) were performed, and a relatively good agreement was observed between the expected and observed outcomes. The AUC values of the nomogram at 3, 5 and 8 years OS were all over 0.780, and the C-index value was 0.746 (Figure 7(A2)). The AUC values of the nomogram at 3, 5 and 8 years CSS were all over 0.900, and the C-index value was 0.870 (Figure 7(B2)). The AUC values of the nomogram at 3, 5 and 8 years PFS were all over 0.790, and the C-index value was 0.827 (Figure7(C2)). Those results showed that our predictive models have good performance in prognostic prediction, especially in CSS.

## 4. Discussion

Currently, tumour development and progression are recognised as an extremely complicated process and should be attributed to massive genetic abnormality rather than to just one or several factors. Therefore, the comprehensive analysis based on gene sets (or signal pathways) is gaining more and more attention. In this study, we found that multiple gene sets were scored differently in non-tumour and TCBC samples, and significantly associated with invasion, progression or tumour grade, which means that the occurrence and development of TCBC may be regulated by multiple signalling pathways (Figure 1). Among these pathways, the UPR signalling was noticed by us for its better relationship with the clinic-pathological characteristics of TCBC.

Previous studies have proven that the UPR signalling has important roles in BC, and the functions of many UPR-related genes in BC have also been revealed one after another. For instance, Nawroth et al. found that silence of EIF4EBP1 expression could reduce the BC cell proliferation [45]. IMP3 protein was expressed higher in the tumour tissues compared to the benign tissues and associated with the tumour stage, tumour grade and prognosis of BC [46]. ATF3 was down-regulated in BC tissues and negatively correlated with tumour stage, and could suppress BC cell metastasis through the upregulation of GSN-mediated actin remodelling [47]. SEC11A mRNA expression was higher in the BC tissues than in the normal bladder, and the cell growth and invasiveness could be inhibited by SEC11A down-regulation [48]. Knockdown of SHC1 could alleviate the cancer-promoting effect of DEPDC1B on BC [49]. In our analysis, EIF4EBP1, IMP3, ATF3, SEC11A and SHC1 also exhibited different expression between BC and non-tumour samples, and were significantly associated with the tumour type, invasive progression, or tumour grade (Supplementary Figure S2). Therefore, the researches above further support the reliability of our results. However, the roles of many other UPR-related genes (e.g. EIF4A1, EIF2S1, SERP1, etc.) in BC are still unclear, and further study of their underlying mechanism is warranted. In addition, as a collection of genes, the impact of UPR on the progression and prognosis of BC remains unclear. Through clinical analysis, we found that the UPR score was higher in tumour samples and significantly associated with tumour grade, invasion and tumour progression (Figure 2). Besides, the high UPR score predicted poor OS, CSS and PFS of TCBC patients, and the prognostic impact of the UPR signalling was partially validated in two external cohorts (Figures 3 and 5). These results indicated that the UPR signalling may act as a risk factor in the tumorigenesis, progression and prognosis of TCBC.

The importance of immune infiltration in BC has been confirmed by numerous studies [50–53], and the UPR pathway also performed crucial functions in immune response [21–24]. Kemp et al. proved that the UPR was critical for the development, proliferation, activation, differentiation and survival of T cells [54]. As an important part of the UPR pathway, IRE1 was found to be activated at the double-positive stage of T cell development and down-regulated in the maturation of CD4 T cell [55]. In the ovarian cancer microenvironment, IRE1α could suppress the mitochondrial activity in CD4 T cells and increase T cell infiltration [56]. In addition, the activated UPR pathway could attenuate the sensitivity of human hepatocellular carcinoma cells (HCC) to natural killer cells [57]. However, the role of UPR in the immune regulation of TCBC has not been studied. To explore this question, we analysed the relationship between the UPR score and various tumour-infiltrating immune cells. The result showed that the UPR score was positively correlated with the infiltration abundance of activated CD4 T cell, type 2 T helper cell, gamma delta T cell and CD56bright natural killer cell (Figure 4). Through external validation analysis, similar results of CD56bright natural killer cell and activated CD4 T cell were also observed (Figure 5). However, the correlation coefficient values between the UPR score and most tumour immune cells were lower than 0.50. Therefore, we thought that the UPR pathway may be involved in regulating the infiltration of certain immune cells, but further researches and validations are needed to confirm this.

Based on the UPR score and clinical indicators, we constructed prognostic nomograms for OS, CSS and PFS (Figure 6). These models exhibited great performance in prognostic prediction of TCBC patients, especially in CSS (AUC values > 0.900; C-index = 0.870) (Figure 7), although there is a great deal of missing clinical information (e.g. performance status, lymphovascular invasion, tumour size, tumour number, treatment, etc.). Therefore, our models may help guide clinicians to make prognosis assessment and treatment decision-making. Surely, the performance of our models still needs to be further validated in the independent external datasets.

By way of conclusion, the UPR pathway was significantly associated with the carcinogenesis, progression and prognosis of TCBC patients. Besides, the UPR pathway may be involved in regulating the infiltration of tumour immune cells. These results may help us further understand the underlying pathological mechanism of TCBC. Moreover, the predictive models we constructed showed robust performance for predicting TCBC prognosis, which may provide a more effective and reliable guide to prognosis assessment and treatment decision-making in the clinic.

## Supplementary Material

Supplemental Material

## Data Availability

The datasets analysed in this study are available in GEO public databases.
