# Supplementary material for: Effect of unfolded protein response on the immune infiltration and prognosis of transitional cell bladder cancer
Source: Ann Med. 2021 Jun 30;53(1):1049–59. doi: 10.1080/07853890.2021.1918346 (PMC8253203; doi:10.1080/07853890.2021.1918346)
Supplement: Supplemental Material [file IANN_A_1918346_SM8864.zip › suppl_data/Supporting legends.docx]

# Supporting legends

**Figure S1.** Differential detection of 50 hallmark gene sets in different tissue types, including normal bladder mucosae, bladder mucosae surrounding cancer, TCBC, and recurrent tumor samples. TCBC，transitional cell bladder cancer; **, p-value < 0.01.

**Figure S2.** Differential detection of UPR-related genes in different tumor types, muscle invasive progression, mon-muscle invasive progression, and tumor grades. NMIBC, non-muscle invasive bladder cancer; MIBC, muscle invasive bladder cancer; Down, low score in TCBC and recurrent tumor samples; Up, high score in TCBC and recurrent tumor samples; **^§^**, p-value < 0.05 in NMIBC and MIBC; **^#^**, p-value < 0.05 in patients with and without muscle invasive progression; ^&^, p-value < 0.05 in patients with and without non-muscle invasive progression; *, p-value < 0.05 in low and high tumor grades.

**Figure S3.** (**A**) Relationship between EIF4EBP1 and different tissue types; (**B**) Relationship between EIF4EBP1 and different tumor types; (**C**) Relationship between EIF4EBP1 and tumor grade. NBM, normal bladder mucosae; BMSC, bladder mucosae surrounding cancer; TCBC, transitional cell bladder cancer; RNMIT, recurrent non-muscle invasive tumor; NMIBC, non-muscle invasive bladder cancer; MIBC, muscle invasive bladder cancer; *, p-value < 0.05; **, p-value < 0.01; ***, p-value < 0.001.

**Table S1.** The clinicopathological information and UPR score of GSE13507.

**Table S2.** The clinicopathological information and UPR score of GSE5287.

**Table S3.** The clinicopathological information and UPR score of GSE1827.
