# Supplementary figures and images for: Effect of unfolded protein response on the immune infiltration and prognosis of transitional cell bladder cancer
Source: Ann Med. 2021 Jun 30;53(1):1049–59. doi: 10.1080/07853890.2021.1918346 (PMC8253203; doi:10.1080/07853890.2021.1918346)

Figure S1

Heatmap of hallmark gene sets

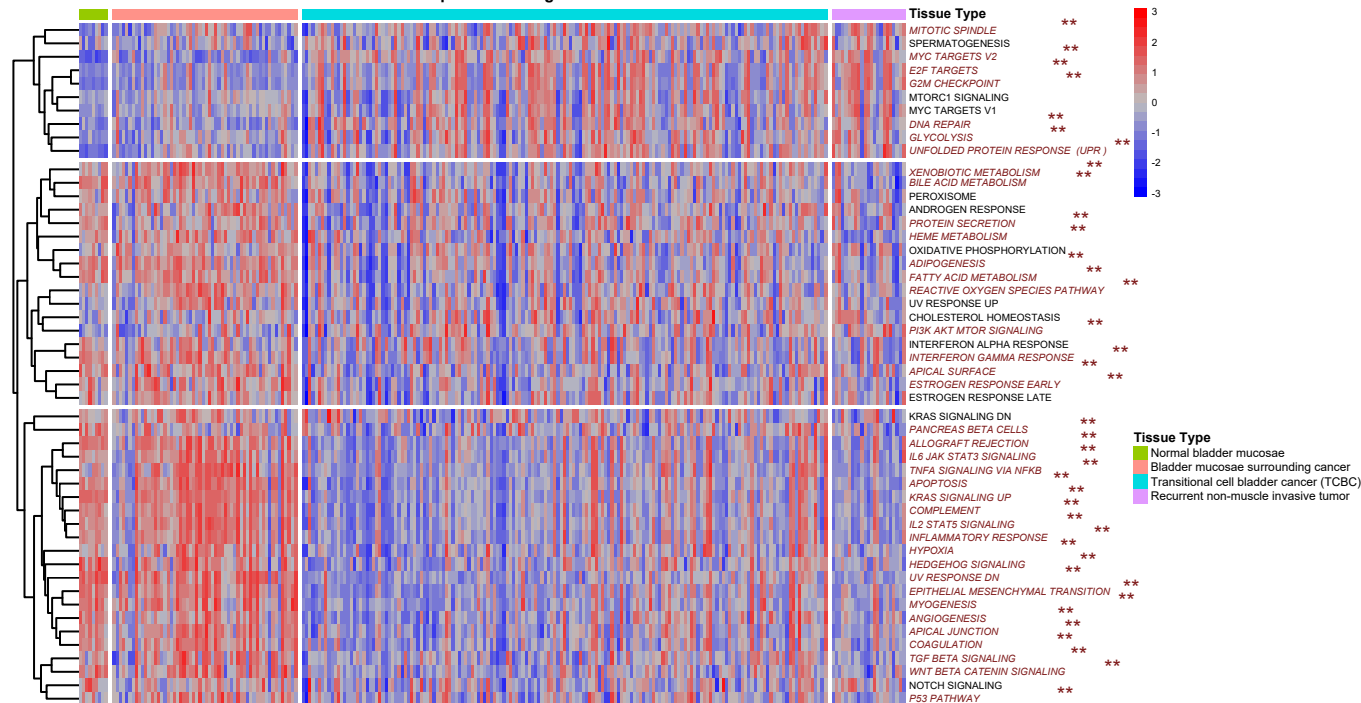

Supplement: Supplemental Material [file IANN_A_1918346_SM8864.zip › suppl_data/FigS1.pdf]

Figure S2

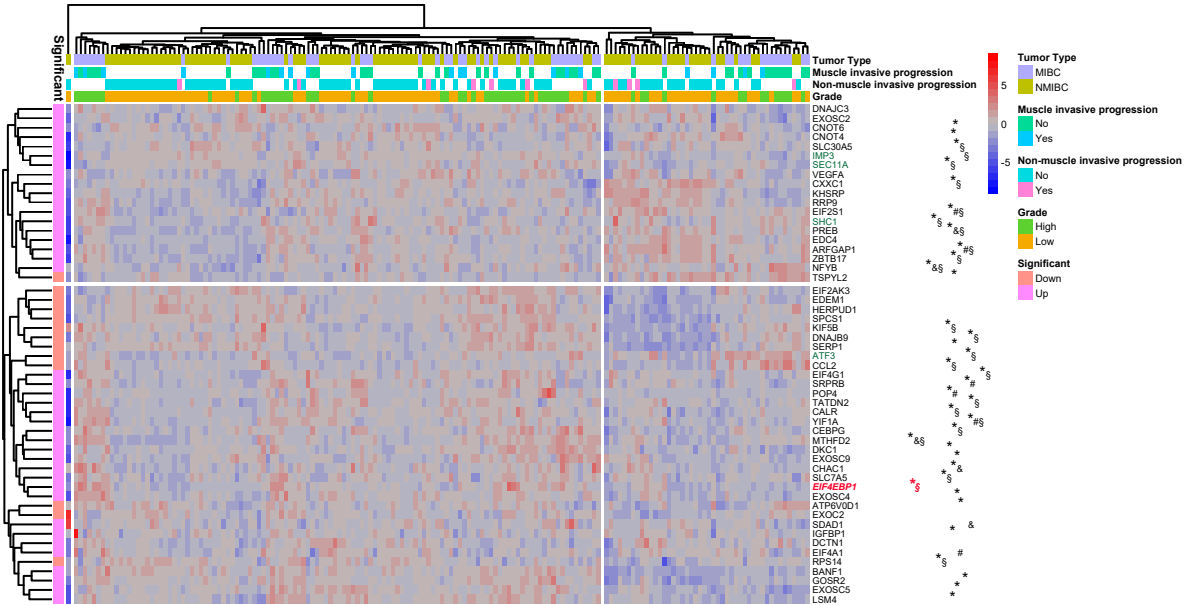

Heatmap of 53 genes in UPR gene set

Supplement: Supplemental Material [file IANN_A_1918346_SM8864.zip › suppl_data/FigS2.pdf]

Figure S3

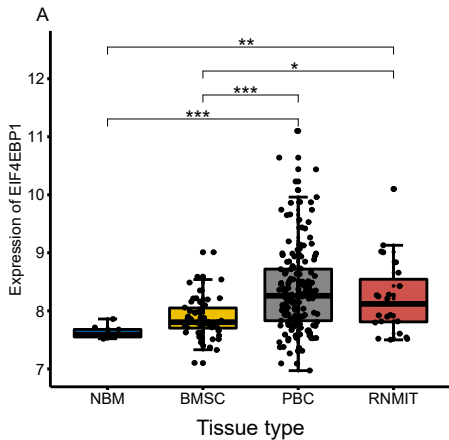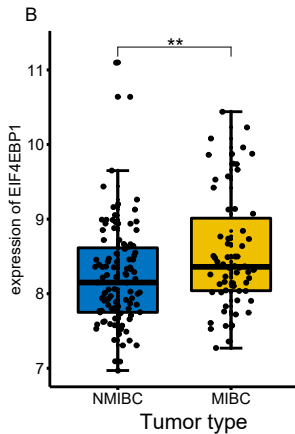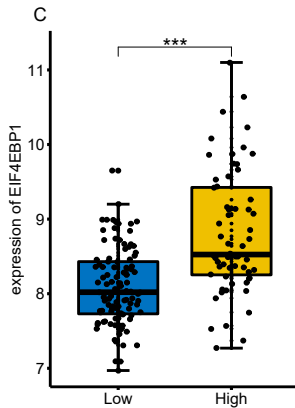

Supplement: Supplemental Material [file IANN_A_1918346_SM8864.zip › suppl_data/FigS3.pdf]
